# Thrombin and factor Xa link the coagulation system with liver fibrosis

**DOI:** 10.1186/s12876-018-0789-8

**Published:** 2018-05-08

**Authors:** Ameet Dhar, Fouzia Sadiq, Quentin M. Anstee, Adam P. Levene, Robert D. Goldin, Mark R. Thursz

**Affiliations:** 1 Department of Surgery and Cancer, Imperial College London, St Mary’s Hospital Campus, London, W2 1NY UK; 2 Institute of Cellular Medicine, Newcastle University, The Medical School, Framlington Place, Newcastle-upon-Tyne, NE2 4HH UK; 3 Department of Histopathology, Imperial College London, St Mary’s Hospital Campus, London, W2 1NY UK

**Keywords:** Fibrosis, Hepatic stellate cell, Thrombin, Factor Xa, Anticoagulation

## Abstract

**Background:**

Thrombin activates hepatic stellate cells via protease-activated receptor-1. The role of Factor Xa (FXa) in hepatic fibrosis has not been elucidated. We aimed to evaluate the impact of FXa and thrombin in vitro on stellate cells and their respective inhibition in vivo using a rodent model of hepatic fibrosis.

**Methods:**

HSC-LX2 cells were incubated with FXa and/or thrombin in cell culture, stained for αSMA and relative gene expression and gel contraction calculated. C57BL/6 J mice were administered thioacetamide (TAA) for 8 weeks with Rivaroxaban (*n* = 15) or Dabigatran (n = 15). Control animals received TAA alone (n = 15). Fibrosis was scored and quantified using digital image analysis and hepatic tissue hydroxyproline estimated.

**Results:**

Stellate cells treated with FXa and thrombin demonstrated upregulation of procollagen, TGF-beta, αSMA and significant cell contraction (43.48%+/− 4.12) compared to culturing with FXa or thrombin alone (26.90%+/− 8.90, *p* = 0.02; 13.1%+/− 9.84, *p* < 0.001). Mean fibrosis score, percentage area of fibrosis and hepatic hydroxyproline content (2.46 vs 4.08, *p* = 0.008; 2.02% vs 3.76%, *p* = 0.012; 276.0 vs 651.3, *p* = 0.0001) were significantly reduced in mice treated with the FXa inhibitor compared to control mice. FXa inhibition was significantly more effective than thrombin inhibition in reducing percentage area of fibrosis and hepatic hydroxyproline content (2.02% vs 3.70%,*p* = 0.031; 276.0 vs 413.1,*p* = 0.001).

**Conclusions:**

FXa promotes stellate cell contractility and activation. Early inhibition of coagulation using a FXa inhibitor significantly reduces TAA induced murine liver fibrosis and may be a viable treatment for liver fibrosis in patients.

## Background

The interaction between the coagulation cascade and liver injury is multifaceted [1]. Although prolongation of the prothrombin time is a well-recognized consequence of chronic liver disease, recent studies demonstrate that patients with advanced fibrosis can also have reduced levels of protein C and antithrombin, balancing any reduction in thrombin generation [2, 3]. This may in part explain the paradoxical increased relative risk of venous thromboembolic disease observed in patients with chronic liver disease in a recent large case-control population based study [4].

Evidence now suggests a role for coagulation proteins in the pathogenesis of liver fibrosis [5]. Firstly, epidemiological studies have demonstrated that prothrombotic conditions promote liver fibrosis [6–9]*.* Secondly, Tissue Factor and fibrin have been shown to be upregulated within fibrotic livers, which is in keeping with a role for vascular dysfunction in fibrogenesis [10–13]. Thirdly, in addition to its role in activating fibrinogen, thrombin has been shown to mediate the cellular activation of macrophages, platelets and hepatic stellate cells (HSCs) via cleavage of the protease-activated receptor, PAR-1 [14, 15] and polymorphisms in the PAR-1 gene have been shown to influence rates of hepatic fibrosis [16]. Finally, warfarin and experimental thrombin antagonists have demonstrated some anti-fibrotic properties in animal models of liver fibrosis [17, 18]. Recently FXa, a protease activated upstream of thrombin in the coagulation cascade, has been shown to promote fibrogenesis via direct PAR receptor cleavage in pulmonary fibrosis [19, 20]. Activation of FXa may therefore drive hepatic fibrogenesis both through direct PAR activation and by thrombin generation, making it an attractive therapeutic target. To date no study has examined the direct effects of FXa on HSC activity. In lung fibrosis, which is biologically analogous to hepatic fibrosis, treatment with a direct inhibitor of FXa reduces fibrosis significantly in a murine bleomycin-inhalation model [20]. An understanding of the effect of FXa on HSCs and fibrogenesis, above and beyond its role in generating thrombin is therefore needed, and the effects of direct FXa inhibition in the setting of hepatic fibrosis need to be evaluated to assess whether it could offer additional efficacy over direct thrombin inhibition as an anti-fibrotic agent.

Using the human LX2 hepatic stellate cell culture line we sought to determine the relative efficacy of FXa and thrombin on HSC activity in vitro. We then evaluated the anti-fibrotic potential in vivo of both direct FXa inhibition with Rivaroxaban (Bayer Healthcare, Germany) and direct thrombin inhibition with Dabigatran, (Boehringer Ingelheim Pharma, Germany) using a murine model of liver fibrosis.

## Methods

### In vitro studies

#### HSC LX2 cell culture

LX-2 cells are a commonly used hepatic stellate cell (HSC) line [21] and were kindly provided by S Friedman (Mount Sinai, New York, New York). Cells were maintained in DMEM (ThermoFisher, UK) supplemented with 10% FBS and 2 mM L-Glutamine. For experimental procedures, cells were incubated for 24 h in a humidified environment of 5% CO_2_ with serum free media alone (control) or serum free media supplemented with 0.5 U/ml FXa (Enzyme Research Lab, USA); 10 nM thrombin (Sigma, UK); or 0.5 U/ml FXa and 10 nM thrombin. FXa at 0.5 – 1 U/ml has been previously used in other studies [22]. After incubation the media was gently removed and cells were fixed by pipetting paraformaldehyde into each well and incubating at room temperature. Cells were immunostained for αSMA, a marker of HSC activation, using a mouse monoclonal antibody raised against alpha-smooth muscle actin (Clone 1A4, Dako Inc., USA). For gene expression studies, total RNA was isolated from cells stimulated with FXa and/ or Thrombin (RNeasy Mini kit, Qiagen Ltd., Crawley, UK), reverse transcribed (RETROscript kit; Thermofisher, UK) and quantitative polymerase chain reaction (qPCR) analysis was performed using TaqMan Gene Expression Assays (ThermoFisher, UK). Data were normalized to the endogenous housekeeping gene GADPH and fold change differences in expression relative to control (untreated LX2) cells were calculated using the Comparative ddCT method [17, 23].

#### HSC gel contractility assay

HSC contractility assays were performed as previously described with some minor modifications [24]. LX-2 cells were layered on top of the collagen lattice in 24 well plates and serum starved for 24 h. Gel contraction was induced by incubation of duplicate wells with 10% FBS, which acted as the positive control, or the proposed agonists, FXa or thrombin in the following combinations: 0.5 U/ml FXa alone; 10 nM thrombin alone; or 0.5 U/ml FXa with 10 nM thrombin. Additional wells containing a cell free lattice and wells containing a lattice with LX2 cells without any agonist, acted as controls for spontaneous gel shrinkage. Gels were released from wall using a micro-pipetting tip (representing time point zero) and photographed at 6 h. Surface area of the collagen gels was measured using digital image analysis software, and images were processed using digital image analysis. Relative contraction of the gels was expressed as a percentage of the surface area contraction of each experimental gel in comparison to gels incubated with media and LX2 cells alone.

### Animals studies

All research using live animals was approved by the local ethics committee and carried out in accordance with the Animal (Scientific Procedures) Act 1986. Institutional guidelines were followed for the care and use of animals. Forty five male C57BL/6 J mice, aged 8 weeks old, were purchased from Jackson Laboratories (USA). All mice were housed under standard conditions and treated with TAA to induce liver fibrosis for 8 weeks via drinking water at a dose of 300 mg/l. Animals were randomly allocated to treatment or control groups. Rivaroxaban and Dabigatran were acquired from commercial source. Tablets were crushed and dissolved in water to achieve fine suspension. Two subgroups (*n* = 15 each) were treated, once daily, by oral gavage with either Rivaroxaban, (FXa inhibitor group) at a dose of 40 mg/kg or Dabigatran (thrombin antagonist group) at a dose of 100 mg/kg. Control group mice (n = 15) received no anticoagulation. Doses of anticoagulants were attained to cause equivalent prolongation of coagulation times, including the International Normalised Ratio (INR) to between 1.5 to 2, as measured using a handheld coagulation meter, following blood sampling from animals tail veins and referencing previous dosing and rodent data [25–27]. Gavage volumes were administered based on each individual animal as per a pre-formulated weight/dose chart. At 8 weeks, all surviving animals from each experimental group were culled by intraperitoneal injection of 0.1 ml Pentoject (Pentobarbitone Sodium Ph.Eur. 200 mg/ml, Animal Care Ltd., UK) followed by cervical dislocation and livers harvested.

### Histopathology and digital image analysis

Liver tissue from each animal was fixed in 10% formalin, processed into paraffin wax and sections stained with Haemotoxylin & Eosin (H&E) and Picro-Sirius red, a commonly used stain for collagen and fibrosis [28]. All sections were examined using a light microscope by a histopathologist blinded to the therapy received. The extent of hepatic fibrosis was assessed using a semi-quantitative score in a manner previously described in mouse models of fibrosis [17] and digital image analysis to calculate mean percentage area of fibrosis. Tissue was stained for αSMA, as described above and examined with a light microscope to calculate the mean number of activated stellate cells for each individual animal per field.

### Tissue hydroxyproline measurement

Hydroxyproline content has been described as a surrogate for collagen content in fibrogenesis [29] and was measured using a commercially available kit (BioVision K555-100, USA). Briefly, pre-weighed frozen liver tissue samples were homogenised in water and hydrolysed in 12 N HCl at 120 °C for 3 h, and 10μl of the hydrolysate was evaporated to dryness under vacuum. Chloramine T (100μl) was added and incubated for 5 min at room temperature. Then 100μl of DMAB (4-(dimethylamino)benzaldehyde) reagent was pipetted into each sample and incubated for 90 min at 60 °C. Absorbance was read at 560 nm and total hydroxyproline content in the samples was extrapolated from the standard curve.

### Statistical analysis

Statistical analysis was performed using SPSS v16 (SPSS, USA). Normally distributed continuous variables were compared by Student’s t-test. Variables which were not distributed normally were compared using the non-parametric tests, Mann-Whitney or Kruskall-Wallis. Quantitative data were expressed as mean +/− standard error of mean (SEM), unless otherwise stated. Statistical significance was accepted at *p* < 0.05.

## Results

### In vitro studies

#### LX2 hepatic stellate cell line

**Fig. 1:**
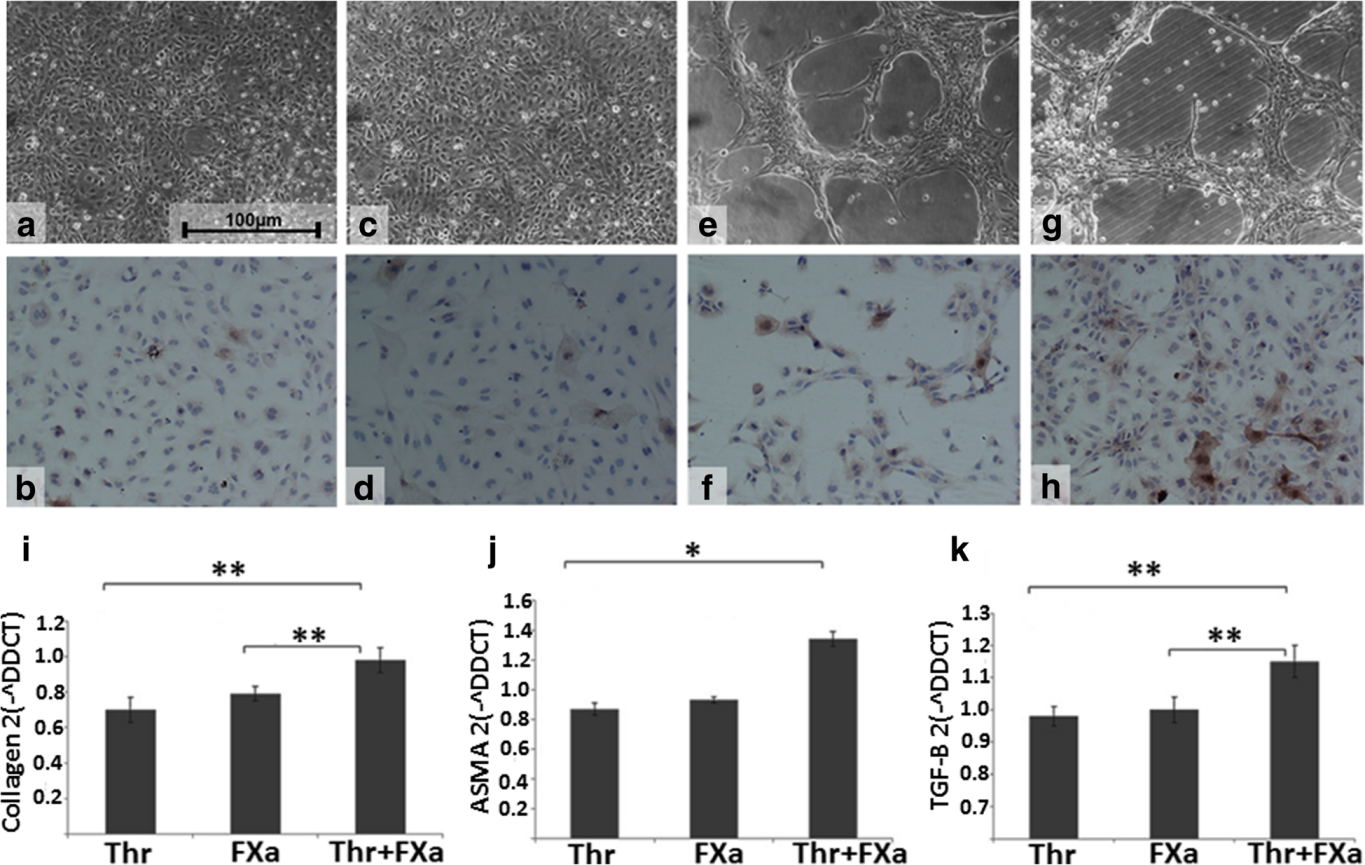
Effect of FXa, thrombin on LX2 cells in vitro. **a** & **b** Incubation of cells with control medium alone resulting in minimal cell contraction and staining for αSMA (× 100 magnification). **c** & **d** Incubation of LX2 cells with thrombin at a dose of 10 nM resulted in some activation of LX2 cells with positive staining for αSMA and some contraction (× 100 magnification) **e** & **f** Incubation of LX2 cells with FXa at a dose of 0.5 U/ml resulted in activation of LX2 cells with positive staining for αSMA and a contracted phenotype (× 100 magnification). **g** & **h** Addition of FXa (0.5 U/ml) in combination with thrombin (10 nM) resulted in activation of LX2 cells with heavy positive staining for αSMA and a contracted phenotype (× 100 magnification). **i** - **k** LX2 cells were incubated with FXa and/or thrombin. αSMA, procollagen and TGF-beta expression were analysed by qRT-PCR. Data were normalized to the endogenous housekeeping gene GADPH and fold change differences in expression relative to control (untreated LX2) cells were calculated using the Comparative ddCT method. Data are Mean ± SEM from one of the representative experiments. Significance was denoted as *p < 0.05, **p < 0.01. Abbreviations: αSMA, alpha smooth muscle actin; FXa, Factor Xa; TGF-beta, Transforming growth factor beta; Thr, Thrombin

To determine the effect of individual coagulation proteins on the activity of HSCs, the principal cell of hepatic fibrosis, LX2 cell lines were cultured. Expression of αSMA and cell contraction was used as markers of stellate cell activation and is represented in Fig. 1. Incubation of LX2 cells with medium alone on a glass surface resulting in minimal contraction and αSMA expression (Fig. 1a&b). Incubation with thrombin demonstrated weakly positive staining for αSMA in LX2 cells (see Fig. 1c&d). αSMA staining and cell contraction was more prominent with when cells were incubated with FXa (Fig. 1e&f). FXa and thrombin together resulted in heavy staining for αSMA, with cell contraction more exaggerated than with thrombin alone (Fig. 1g&h). Relative gene expression using qPCR demonstrated a significant increase in the procollagen, and TGF expression when LX2 cells incubated with both FXa and Thrombin, compared to thrombin (*p* = 0.01; p = 0.01) or FXa (p = 0.01; *p* = 0.02) alone (Fig. 1i&k). FXa and thrombin resulting in a significant increase in αSMA gene expression compared to thrombin alone (*p* = 0.04) (Fig. 1j). There was no significant difference in procollagen, TGF-beta or αSMA gene expression between cells incubated with FXa or thrombin alone.

### HSC contractility assay

**Fig. 2:**
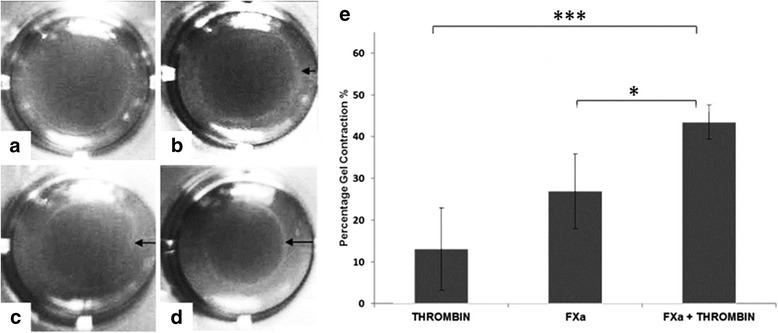
Effect of FXa and thrombin on LX2 hepatic stellate cell contractility assays at 6 h Collagen lattices with LX2 agonists (**a - d**) and bar graph showing percentage gel contraction for FXa and/or thrombin. (**e**). LX2 cell collagen gels incubated with medium alone (**a**) with thrombin (10 nM) (**b** & **e**) with FXa (0.5 U/ml) (**c** & **e**) and with FXa & thrombin (**d** & **e**). Relative contraction of the gels was expressed as a percentage of the surface area contraction of each experimental gel in comparison to gels incubated with media and LX2 cells alone. Significance was denoted as *p < 0.05, ***p < 0.001 Abbreviations: FXa, Factor Xa. *Black arrows indicate shrinkage of gel margin from wall of gel.*

The use of contractility gel assays allows for a quantitative measurement of hepatic stellate cell contractility [24]. Incubation of LX2 cells with medium alone resulted in a small degree of spontaneous gel shrinkage at 6 h (Fig. 2a), and was used as a control. The mean surface area of experimental gels was expressed as a percentage of the surface area of mean control gels and subtracted from 100 to calculate the percentage gel contraction. This allowed for any correction of spontaneous gel shrinkage that may have occurred. Incubation with FBS, which is used as a positive control in this type of assay, resulted in a significant contraction of gels, thus validating the technique. The addition of FXa and thrombin individually resulted in contraction of gels. FXa treated gels demonstrated more contraction at 6 h, than those treated with thrombin (26.90% +/− 8.90 versus 13.10% +/− 9.84), but the difference did not reach significance (Fig. 2b, c & e). The addition of FXa and thrombin together however demonstrated a potentiated effect on cell contraction (43.48% +/− 4.12), with significantly more contraction compared to incubation with FXa (*p* = 0.02) or thrombin alone (*p* < 0.001) (Fig. 2d & e).

### Animal studies

Early mortality affected both treatment and control subgroups equally. Two to three mice were lost from each group due to procedural complications following gavage during the course of the study, and none were related to spontaneous hemorrhage. All animals were weighed every second week. No animal recorded a reduction in overall weight at the end of the study, in comparison to its starting weight.

### Fibrosis scoring and percentage area of fibrosis in TAA treated mice

**Fig. 3:**
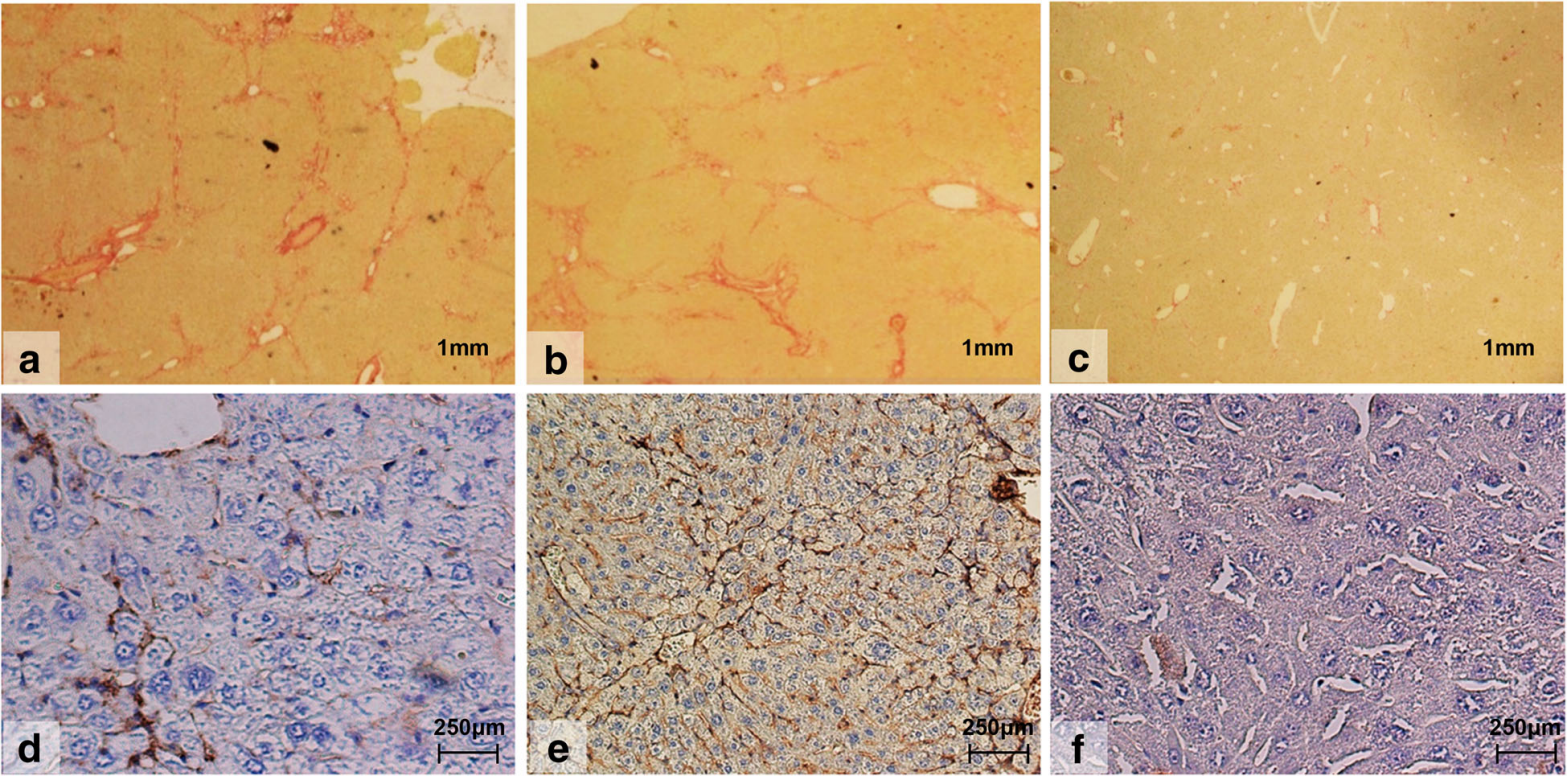
Histological images using Picro-sirius red staining (**a-c**) of liver tissue for fibrosis and immunohistochemical staining for αSMA (**d-f**) in mice treated with 8 weeks of thioacetamide (TAA) with or without FXa or thrombin inhibition. Control mice treated with TAA (*n* = 13) alone (A; × 15magnification). Mice treated with TAA and the thrombin inhibitor (*n* = 12), Dabigatran (100 mg/kg) (B; × 15 magnification). Mice treated with TAA and the FXa inhibitor (n = 13), Rivaroxaban (40 mg/kg) (C; × 15 magnification)

**Fig. 4:**
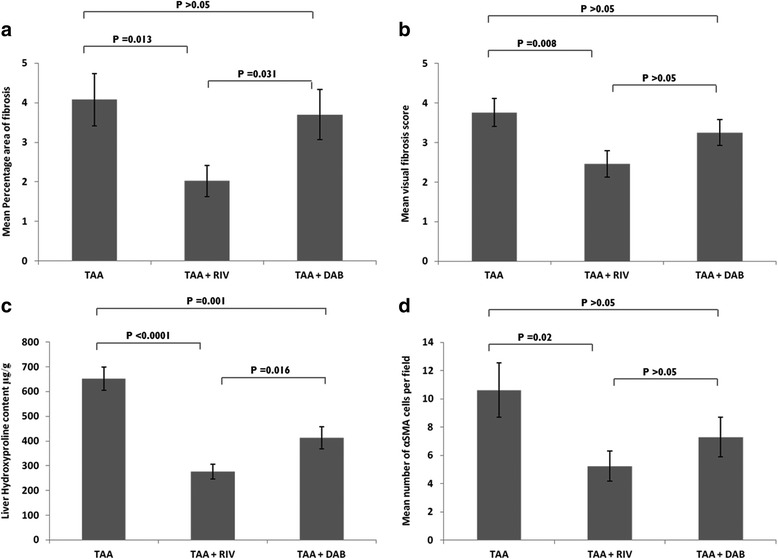
Mean percentage area of fibrosis, mean fibrosis visual scores, liver hydroxyproline and αSMA cells following 8 weeks of exposure with TAA. Bar graphs showing (**a**) mean percentage area of fibrosis. Picro-Sirius red stained histological sections of liver were assessed under light microscopy for fibrosis. The extent of hepatic fibrosis was assessed in field of view (*n* = 10) under a light microscope, using a semi-quantitative score [17] coupled with digital image analyses (**b**) and mean visual fibrosis score agreed by two independent histopathologists using standardized protocols (**c**) liver hydroxyproline content. Frozen liver samples (TAA; n = 13, TAA + RIV; n = 13; TAA + DAB; *n* = 11) were homogenized in water and hydrolysed in HCl. Hydroxyproline was oxidized and reacted with DMAB to produce colour change which is directly proportional to Hydroxyproline concentration and (**d**) mean number of αSMA cells following 8 weeks of TAA exposure. Histological sections were stained for αSMA as described under methods and examined under a light microscope. Number of stellate cells stained positive for αSMA were counted per field of vision (*n* = 6), and an average calculated, which represented the mean number of activated stellate cells. Data are mean +/− SEM unless otherwise stated. Abbreviations: DAB, Dabigatran; RIV, Rivaroxaban; TAA, Thioacetamide**;** DMAB, 4-(dimethylamino) benzaldehyde. All sections were examined with a light microscope and the number of stellate cells stained positive for αSMA were counted per field of vision at × 20 objective. Six fields per section were randomly chosen, and an average calculated, which represented the mean number of activated stellate cells for each individual mouse per field

After 8 weeks of exposure with TAA via drinking water (300 mg/L), control mice demonstrated bridging fibrosis with occasional nodule formation (Fig. 3a). Mice treated with the direct thrombin antagonist, Dabigatran (100 mg/kg), exhibited frequent bridging fibrosis, similar to control mice, but only occasional nodule formation (Fig. 3b). In contrast, mice treated with the direct FXa inhibitor Rivaroxaban (40 mg/kg) demonstrated milder fibrosis, with fibrous expansion around the central veins, less frequent bridging and the absence of nodules (Fig. 3c). In keeping with this finding, mean fibrosis scores in mice treated with Rivaroxaban were significantly reduced (*p* = 0.008) in comparison to control mice (2.46 ± 0.33 versus 3.76 ± 0.36) and there was a significant reduction (*p* = 0.012) in mean percentage area of fibrosis (2.02% ± 0.39 versus 4.08 ± 0.66) (Fig. 4). In contrast, mice treated with Dabigatran, had a mean fibrosis score of 3.25 ± 0.63 (SEM) and mean percentage area of fibrosis of 3.70% ± 0.63 (SEM). This represented only a 13 and 9% reduction respectively compared to control mice and did not reach statistical significance (Fig. 4). When comparing direct FXa inhibition to direct thrombin antagonism, FXa inhibition demonstrated a significant reduction in mean percentage area of fibrosis (*p* = 0.031) (Fig. 4).

### Alpha smooth muscle actin expression in TAA treated mice

Following 8 weeks exposure to TAA, mice treated with Rivaroxaban demonstrated a significant reduction in the mean number of αSMA positive hepatic stellate cells per field of view compared to control group mice (5.23 ± 1.06 versus 10.62 ± 1.92; *p* = 0.02). Treatment with Dabigatran however only resulted in a trend towards decreased reduction in αSMA expression compared to control mice (7.29 ± 1.39 versus 10.62 ± 1.92; *p* > 0.05) (Figs. 3 and 4).

### Hepatic hydroxyproline content in TAA treated mice

Mice treated with Rivaroxaban demonstrated a significant reduction in the mean hepatic hydroxyproline content compared to control group mice (276.0 ± 30.2 versus 651.3 ± 46.7; *p* < 0.0001). Dabigatran (100 mg/kg) resulted in a significant reduction in the mean hepatic hydroxyproline content compared to control group mice (413.1 ± 1.39; *p* = 0.001). FXa inhibition was associated with significantly lower hydroxyproline levels than that observed with thrombin inhibition (*p* = 0.016) (Fig. 4).

## Discussion

Recent evidence suggests hepatic fibrogenesis is associated with prothrombotic tendencies [3, 4, 6, 17] and coagulation proteins are up-regulated in fibrotic livers [10–12]. The presence of activated coagulation proteins in this setting may also act as important mediators in hepatic fibrosis. We sought to examine the effects of FXa and thrombin on HSCs in vitro, and to evaluate the anti-fibrotic potential of both direct FXa and thrombin inhibition using novel anticoagulants in a murine model of fibrosis induced by TAA.

### FXa and thrombin increase stellate cell contractility and activation in vitro

The stellate cell is the principal cell type involved in hepatic fibrogenesis and on stimulation contracts, a state that is characterized by the increased expression of the contractile filament protein, alpha smooth muscle actin [30, 31]. We have demonstrated that culturing LX2 cells with FXa or thrombin independently and in combination results in stellate cell contraction. FXa concentration used (0.5 U/ml or 174 nM) was within the plasma physiological range (~ 150-200 nM). There is an upregulation in procollagen, TGF-beta and αSMA gene expression only when FXa and Thrombin in combination were given. These results are corroborated by our gel contraction assay, in which we were able to quantify a highly significant increase in gel contraction when FXa and thrombin were administered in combination compared to alone. Both the upregulation of procollagen, TGF-Beta, and αSMA and the contraction of LX2 cells in culture and gel contraction assays confirm FXa and thrombin independently potentiate HSC activation and in synergy this is significantly increased*.* A potential mode of action for these proteins can be postulated. Thrombin is known to be a mediator of stellate cell activation [18] and it is now established that the cellular actions of thrombin are in part mediated by PAR signaling [14]. PAR receptors are a family of widely expressed G-protein-coupled receptors, that transduce transmembrane signaling and four PARs have been identified [32]. PAR-1 and -3 are both preferentially activated by thrombin. PAR-4 has reduced affinity and PAR-2 is resistant to thrombin activation. A substantial body of evidence from in vivo and in vitro studies is now accumulating to suggest PAR-1 activation leads to HSC activation [5, 33–35]. Whilst thrombin PAR-1 mediated ligation results in stellate cell activation, the mechanism by which fibroblasts are activated by FXa has been less well studied. FXa, is a coagulation factor generated at the point of convergence of the intrinsic and extrinsic coagulation pathways and responsible for the conversion of prothrombin to thrombin. Recent evidence suggests that FXa activates PAR-1, in a similar fashion to thrombin, but also has been demonstrated to activate PAR-2 [36, 37]. In pulmonary fibrosis which is a well-documented paradigm for liver fibrosis, FXa mediated PAR1 activation has been demonstrated [20]. PAR-2 expression has recently been demonstrated on both hepatic stellate cells [1, 34, 35]: up-regulated in fibrotic livers [1]. PAR-2 deficiency, using PAR-2 knockout mice, has been shown to reduce CCL4 induced liver fibrosis [38]. FXa via the added role of PAR-2 mediated activation could explain the increased effect on stellate cell contraction and activation demonstrated with FXa or FXa with thrombin, compared to thrombin alone. In vivo FXa is also central in converting prothrombin to thrombin, with one molecule of FXa generating a ‘thrombin burst’ of over 1000 thrombin molecules [39]. Therefore FXa is pro-fibrotic by two pathways. Firstly it drives thrombin production and secondly, independent of its pro-coagulant activity, we have demonstrated it potentiates activation of stellate cells, but further experiments are required to confirm if this is via PAR-1 and PAR-2 mediated mechanisms.

### The efficacy of FXa and thrombin inhibition as antifibrotic therapies in vivo

From the pathway described above, we can hypothesize that targeting coagulation proteins earlier in the cascade should enhance the anti-fibrotic effect rather than inhibiting thrombin alone. We sought to study this further by using the oral anticoagulants, Rivaroxaban, a direct FXa inhibitor, and Dabigatran, a direct thrombin antagonist in vivo using mice exposed to 8 weeks of TAA orally to induce liver fibrosis. Preliminary experiments were conducted to adjust the doses in animals to attain the required level of anticoagulation. Tail bleeding was done to ascertain coagulation time to give equivocal prolongation of coagulation time for the doses used. Doses were increased until the same prolongation for both rivaroxaban and dabigatran was achieved. Using this model we have demonstrated a significant reduction in mean percentage area of fibrosis determined by digital image analysis, mean fibrosis scores, αSMA expression and hepatic hydroxyproline content in mice treated with Rivaroxaban, at a dose of 40 mg/kg versus control mice. In contrast, Dabigatran at a dose of 100 mg/kg did not result in a significant reduction in these parameters with the exception of hepatic hydroxyproline, versus control animals and a significantly higher mean percentage area of fibrosis and hepatic hydroxyproline content compared to Rivaroxaban treated mice, despite an equivalent level of anticoagulation. The concordance between our in vitro and in vivo data highlights the particular importance of the coagulation protein FXa, as well as thrombin in hepatic fibrosis. In vivo the administration of the FXa inhibitor, Rivaroxaban, not only inhibits FXa, but will prevent the downstream production of the thrombin ‘burst’ [25]. Accordingly, the significant reduction in fibrosis and αSMA expression seen in mice exposed to TAA and treated with Rivaroxaban, could be explained by both the direct reduction in FXa mediated PAR1 and PAR2 activation, as well as the indirect reduction in thrombin mediated stellate cell activation via PDGF, TGF-beta and PAR1 ligation. This would also explain why direct thrombin inhibition with Dabigatran was less effective anti-fibrotic intervention compared to FXa inhibition with Rivaroxaban, since inhibition of thrombin alone would fail to block FXa PAR mediated activation of stellate cells and fibrogenesis. Parallels can be drawn with previous unpublished data from our group, in which Ximelagatran, a thrombin antagonist that has now been withdrawn from clinical use, was found to be less effective than warfarin in reducing fibrosis in wild-type C57BL/6 J mice, possibly suggesting that modulation of factors II, VII, IX and X by warfarin provided additional anti-fibrotic effects over direct thrombin (II) blockade alone (Anstee and Thursz, un-published data). Heparin will have a similar effect to FXa inhibitors due to a similar mode of action. Therefore any potential benefits demonstrated with Direct-acting Oral anticoagulants could also be demonstrated with heparin, however further studies are required.

## Conclusions

In summary the results generated by this study further support the role of coagulation proteins in the pathogenesis of hepatic fibrogenesis. We have demonstrated that FXa and thrombin both independently and in synergy promote hepatic stellate cell activation and early inhibition of coagulation using an FXa inhibitor significantly reduced the rate of hepatic fibrosis and stellate cell activity in a mouse model of liver fibrosis compared to direct thrombin inhibition. This effect is likely due to the failure of direct thrombin inhibition to block PAR-mediated stellate cell activation by FXa, and highlights the importance of inhibiting the coagulation cascade earlier when planning anti-fibrotic therapies. A UK based phase II study examining the effects of anticoagulation in liver fibrosis in patients post liver transplant infected with HCV is currently being undertaken to further evaluate the anti-fibrotic potential of anticoagulation in liver disease (ISRCTN 12504151). In view of our findings and recent trials demonstrating the safety of FXa inhibitors, consideration should be given to trials using these new agents as potential anti-fibrotics which, unlike warfarin, do not require frequent monitoring.

## Abbreviations

BSA: Bovine serum albumin
DMEM: Dulbecco’s minimal essential medium
FBS: Foetal bovine serum
FXa: Factor Xa
HCV: Hepatitis C virus
HSC: Hepatic stellate cells
PAI-1: Plasminogen activator inhibitor-1
PAR: Protease-activated receptor
PBS: Phosphate buffered saline
PDGF: Platelet derived growth factor
TAA: Thioacetamide
TF: Tissue factor
TGF-beta: Transforming growth factor beta
αSMA: Alpha smooth muscle actin
